# Therapeutic potential of mesenchymal stem cells overexpressing human forkhead box A2 gene in the regeneration of damaged liver tissues

**DOI:** 10.1111/j.1440-1746.2012.07137.x

**Published:** 2012-08

**Authors:** Jong-Woo Cho, Chul-Young Lee, Yong Ko

**Affiliations:** * Department of Biotechnology, Korea UniversitySeoul, Korea; † Department of Animal Material Engineering, College of Science and Natural Resource, Gyeongnam National University of Science and TechnologyJinju, Korea

**Keywords:** forkhead box A2 gene, hepatocyte, mesenchymal stem cell, stem cell therapy

## Abstract

**Background and Aim:**

Although a liver transplantation is considered to be the only effective long-term treatment in many cases of liver diseases, it is limited by a lack of donor organs and immune rejection. As an autologous stem cell approach, this study was conducted to assess whether forkhead box A2 (*Foxa2*) gene overexpression in bone marrow-derived mesenchymal stem cells (MSC) could protect the liver from hepatic diseases by stimulating tissue regeneration after cell transplantation.

**Methods:**

Rat MSC (rMSC) were isolated, characterized, and induced to hepatocytes that expressed liver-specific markers. Four different treatments (control [phosphate-buffered saline], rMSC alone, rMSC/pIRES–enhanced green fluorescent protein (EGFP) vector, and rMSC/pIRES–EGFP/human *Foxa2*) were injected into the spleen of carbon tetrachloride-injured rats. Biochemical and histological analyses on days 30, 60, and 90 post-transplantation were performed to evaluate the therapeutic capacities of MSC overexpressing *hFoxa2*.

**Results:**

rMSC transfected with *hFoxa2* were induced into hepatogenic linage and expressed several liver-specific genes, such as, *Foxa2*, α-fetoprotein, cytokeratin-18, hepatocyte nuclear factor-1α, and hepatocyte growth factor. A group of animals treated with MSC/*hFoxa2* showed significant recovery of liver-specific enzyme expressions to normal levels at the end of the study (90 days). Furthermore, when compared to the fibrotic areas of the samples treated with MSC alone or MSC/vector, the fibrotic area of the samples treated with rMSC/*hFoxa2* for 90 days significantly decreased, until they were completely gone.

**Conclusions:**

Human *Foxa2* efficiently promoted the incorporation of MSC into liver grafts, suggesting that *hFoxa2* genes could be used for the structural or functional recovery of damaged liver cells.

## Introduction

The liver is one of the most important organs in the human body. Although it has considerable inherent regenerative capacity,^1^ a liver transplantation is considered to be the only effective long-term treatment in many cases of liver disease. Nevertheless, liver transplantation is limited by a lack of donor organs and immune rejection. As an alternative, strategies using various types of stem cells and their progenies have recently been utilized in animal models of chronic diseases.^2^,^3^

Bone marrow is a major source of hematopoietic multipotent stem cells, as well as mesenchymal stem cells (MSC).^4^,^5^ Owing to the ease of cell isolation and the rare possibility of tumorigenesis, bone marrow has commonly been used to treat leukemia.^6^ In addition, a number of studies have shown that bone marrow-derived MSC are competent to differentiate into osteoblasts, chondrocytes, adipocyte, and hepatocyte-like cells.^7^^–^^9^ However, as there has been limited success with the use of MSC for the treatment of chronic liver diseases in animal studies, other approaches are needed. One of these is to use MSC that overexpress liver-specific regulatory factors. One candidate gene is the forkhead box (*Fox*) gene family.

This gene family includes more than 100 genes, and is characterized by the presence of a winged-helix DNA-binding domain, with a sequence that is conserved from yeast to humans.^10^,^11^ Specifically, hepatocyte nuclear factor-3 (HNF-3) (*Foxa*) genes are transcription factors that play important roles in the development of the foregut endoderm, such as the lung, thyroid, and pancreas.^12^,^13^ The *Foxa* genes include three subfamilies of HNF-3α (*Foxa1*), HNF-3β (*Foxa2*), and HNF-3γ (*Foxa3*) genes,^14^ and *Foxa2* is a liver transcription factor that regulates the development of liver organogenesis, as well as the expression of liver-specific genes of α1-antitrypsin, albumin, and transthyretin.^15^,^16^ In addition, it has been reported that *Foxa2* expression is required for insulin signaling, as well as other liver metabolisms,^17^ which has made it a primary target gene in the treatment of liver-related diseases.^18^

Several studies of various factors associated with liver regeneration have been reported;^19^^–^^21^ however, as an autologous stem cell approach, the effects of MSC overexpressing a specific transcription factor on the recovery of damaged liver tissue have not been extensively explored. Thus, this study was conducted to verify whether human *Foxa2* (*hFoxa2*) overexpression in rat MSC (rMSC) could protect the liver from hepatic diseases by stimulating tissue regeneration after cell transplantation.

## Methods

**Isolation and culture of rMSC.** The present study was conducted following approval by the Korea University Institutional Animal Care and Use Committee. Bone marrow was collected from 4∼6 week old Sprague–Dawley (SD) rats, and MSC were prepared using Ficoll (GE Healthcare, Little Chalfont, Buckinghamshire, UK). The harvested cells were incubated with either a mouse antirat CD34 antibody (1:500; Santa Cruz Biotechnology, Santa Cruz, CA, USA) or a mouse antirat CD90 antibody (1:500; AbD Serotec, Oxford, UK) at 4°C for 30 min. CD90^+^ cells were isolated by fluorescence-activated cell sorting (FACS) and cultured in high glucose Dulbecco's modified Eagle's medium containing 10% fetal bovine serum and 1% penicillin/streptomycin (Invitrogen, Carlsbad, CA, USA) at 37°C and 5% CO_2_. At 2 days after seeding, the medium was changed to remove non-adherent cells, and CD90^+^cells were refed every 3–4 days thereafter. rMSC between the third and fifth passages were used in this study.

***In vitro* differentiation of rMSC.** rMSC were induced into multiple lineages in induction media for 4 weeks. Induction media for adipogenic differentiation contained 0.5 µM 3-isobutyl-1-methylxanthine, 200 µM indomethacin, 10 µM insulin, and 1 µM dexamethasone (DEX), while that for osteogenic differentiation contained 0.1 µM DEX, 10 mM β-glycerophosphate, and 50 mg L-ascobic acid, and that for hepatogenic differentiation contained 10 ng/mL recombinant human fibroblast growth factor-4 and 20 ng/mL rh hepatocyte growth factor (HGF). In addition, differentiated hepatocytes were changed to a maturation medium (25 ng/mL oncostatin M and 1 µM DEX) and cultured for an additional 3 weeks. Cells were then fixed in cold 4% paraformaldehyde for 1 h, after which they were stained with Oil Red O for adipogenic differentiation and with Tris-maleate solution for osteogenic differentiation. Furthermore, differentiation was confirmed by reverse transcription–polymerase chain reaction (RT–PCR) analyses using the primers described in Table 1. Hepatogenic differentiation was confirmed by Western blot analysis of albumin expression.

**Table 1:** Polymerase chain reaction primer sequences

Gene	Sequence	Annealing temperature (°C)	Product size (bp)
PPAR-γ2	Forward: 5′-GAG CAT GGT GCC TTC GCT GA Reverse: 5′-AGC AAG GCA CTT CTG AAA CCG A	52.5	564
Osteopontin	Forward: 5′-GCT CTA GAG CAC AAT CTT CTA GCC CC Reverse: 5′-GAC GTC GAC TGA CCT CAG TCC GTA AGC C	53	319
HNF-3β	Forward: 5′-AGC AGC AAC ATC ATC ACA GC Reverse: 5′-AAA GTT CCC CCA ATG TTT CC	52.5	313
CK-18	Forward: 5′-TCA AGA ACT GGG GCA CTA CC Reverse: 5′-CAT GTC TTT GCT GGC TTC AA	52	383
CK-19	Forward: 5′-TCC TCC TCA CCA TGA CTT CC Reverse: 5′-CGA ATC TTC ACC TCC AGC TC	55	358
Albumin	Forward: 5′-CTT CAA AGC CTG GGC AGT AG Reverse: 5′-AGT AAT CGG GGT GCC TTC TT	55	395
AFP	Forward: 5′-TGG AGA AGT GCT CCC AGT CT Reverse: 5′-GCA GTG GTT GAT ACC GGA GT	57.5	359
HGF	Forward: 5′-ACA CAT CTG TGG GGG ATC AT Reverse: 5′-TGG TGC TGA CTG CAT TTC TC	55.5	396
c-Met	Forward: 5′-TGT GCA TTC CCC ATC AAA TA Reverse: 5′-CAC AGG ATA GGA ACC CAG GA	51	373
MMP-2	Forward: 5′-GGA CAG TGA CAC CAC GTG AC Reverse: 5′-TCC AGT TAA AGG CAG CGT CT	55	240
GAPDH	Forward: 5′-AGA CAG CCG CAT CTT CTT GT Reverse: 5′-TAC TCA GCA CCA GCA TCA CC	56	323

AFP, α-fetoprotein; CK-18, cytokeratin-18; CK-19, cytokeratin-19; HGF, hepatocyte growth factor; HNF-3β, hepatocyte nuclear factor-3β; MMP-2, matrix metalloprotease-2; PPAR-γ2, peroxisome proliferator-activated receptor-γ2.

**Transfection of cells.** The rat *Foxa2* gene (*rFoxa2*; GenBank NM_012743) and *hFoxa2* gene (GenBank BC011780) were cloned into a pIRES–enhanced green fluorescent protein (pIRES–EGFP) vector (Clontech, Mountain View, CA, USA). pIRES–EGFP only, pIRES–EGFP/*rFoxa2*, or pIRES–EGFP/*hFoxa2* was transfected into rMSC using lipofectamine (Invitrogen, USA), according to the manufacturer's instructions. Geneticin (G418) (Gibco, Gaithersburg, MD, USA) was added to select stably-transfected cells. Cells were then refed with selective medium containing 400 µg G418 per mL every 3 days for 2 weeks. Stably-transfected cells were confirmed based on both fluorescence emission under a fluorescent microscope (Carl-Zeiss, Goettingen, Germany) and by RT–PCR for the presence of *Foxa2* expression (Table 1).

**RNA isolation and RT–PCR analysis.** Total RNA was extracted from transfected rMSC using Trizol (Gibco, USA). A total of 2 µg total RNA was used to perform RT–PCR with various primers (Table 1) using RT-PreMIX (BIONEER, Daejeon, Korea).

**Western bolt analysis.** Cytosolic fractions were prepared from rMSC using protein extraction buffer (Invitrogen, USA). Protein extracts were separated by 10% sodium dodecylsulfate–polyacrylamide gel electrophoresis under denatured conditions, and then transferred to a polyvinylidene difluoride membrane. After the membrane was blocked with 5% skim milk, rabbit anti-albumin primary antibody (1:1000; Abcam, Cambridge, UK) was applied, and the samples were incubated at 4°C for 24 h, after which a horseradish peroxidase-labeled antirabbit secondary antibody (1:1000; Abcam, UK) was applied, and the samples were incubated at 37°C for 1 h. The intensity of each band was visualized by an enzyme-linked chemiluminescence imaging system (GE Healthcare, UK).

**Immunocytochemistry.** Fluorescence staining was conducted on 12-well plates (BD Biosciences, San Jose, CA, USA), in which 3∼4 × 105 rMSC/well were plated. Differentiated hepatocytes were fixed with 4% paraformaldehyde at 4°C for 30 min. The cells were then washed three times with phosphate-buffered saline (PBS) and incubated with albumin primary antibody (1:1000; Santa Cruz, USA) at 4°C for 24 h. Cells were subsequently incubated with rabbit antigoat immunoglobulin–phycoerythrin secondary antibody (1:1000; Santa Cruz, USA) at 37°C for 1 h, followed by counterstaining with 4′,6′-diamidino-2-phenylindole dihydrochloride solution. Albumin expression was determined using a fluorescence microscope.

**Carbon tetrachloride-induced liver disease model and rMSC transplantation.** Liver fibrosis was induced by an intraperitoneal injection of carbon tetrachloride (CCl4; Sigma, St Louis, MO, USA) mixed with olive oil (1:1 by volume) into recipient SD rats at a dose of 7.78 µM/kg (or 1.5 mL/kg) body weight, twice per week for 8 weeks. The same volume of olive oil alone was injected into the control group. For cell transplantation, four different treatments (control [PBS], rMSC, rMSC/pIRES–EGFP, and rMSC/pIRES–EGFP/*hFoxa2*) were injected into the spleen of CCl4-injured rats. The rats were killed on days 30, 60, and 90 post-transplantation, after which liver tissues were extracted for analyses.

**Histological analysis of the liver.** Liver specimens were fixed with 4% paraformaldehyde and embedded in paraffin. Tissue sections were cut into 4-um sections, and then analyzed by hematoxylin–eosin (HE) staining for the recovery of fibrosis and by immunohistochemical staining for the expression of α-smooth muscle actin (α-SMA) (1:200, Invitrogen; USA)

**Biochemical analyses of sera.** Sera collected from each treatment group were prepared and analyzed for the expression of several hepatic enzymes, such as aspartate aminotransferase (AST), alanine aminotransferase (ALT), alkaline phosphatase (ALP), bilirubin, and lactate dehydrogenase (LDH), using a TBA-200FR NEO analyzer (Toshiba, Tokyo, Japan).

**Stastical analyses.** Three independent trials of serum analyses and Western blots were conducted, and the resulting data were analyzed by Student's *t*-test using SPSS (version 12.0; SPSS, Chicago, IL, USA) and SigmaPlot (version 10.0; Systat Software, San Jose, CA, USA). *P*-values less than 0.05 were considered to be statistically significant.

## Results

**Figure 1:**
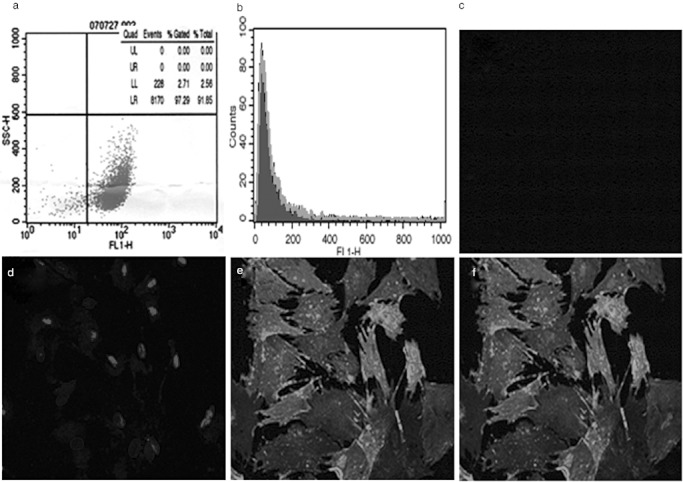
Characterization of isolated rat mesenchymal stem cells (rMSC) by flow cytometric analysis. Fluorescence-activated cell sorting (FACS) analyses of rMSC with anti-CD90 antibody (a) and with anti-CD34 antibody (b). Immunocytochemical analyses of rMSC with CD34 antibody (c), 4′,6′-diamidino-2-phenylindole dihydrochloride (DAPI) (d), and CD90 antibody (e). Merged image of DAPI with CD90 (f).

**Figure 2:**
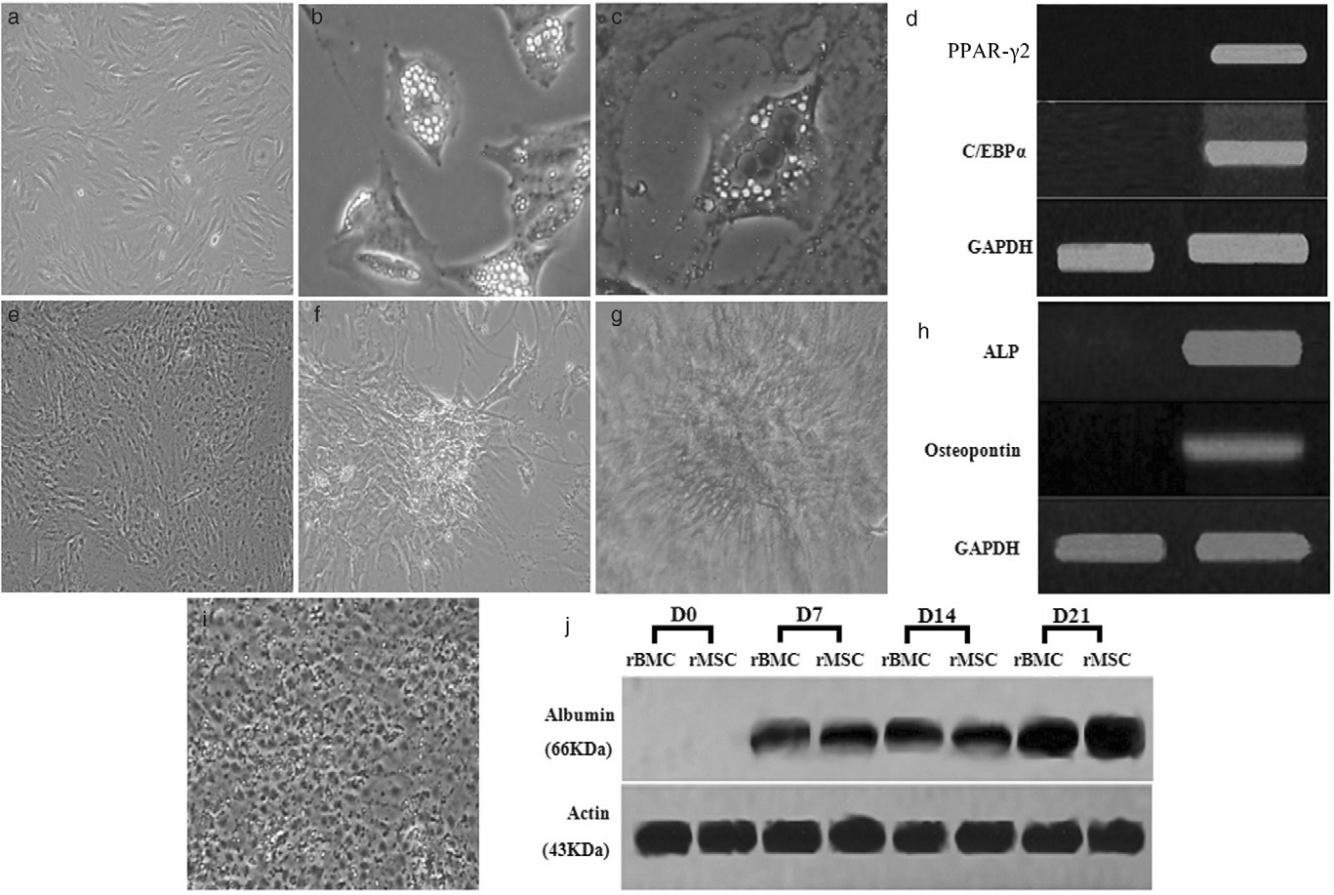
Differentiation of rat mesenchymal stem cells (rMSC) into adipocytes, osteocytes, and hepatocytes. Undifferentiated rMSC (control) (a,e). Morphologies of adipocytes differentiated (b) and stained by Oil Red O (c). Morphologies of osteocytes differentiated (f) and stained by alkaline phosphatase (g). Peroxisome proliferator-activated receptor-γ2 (PPAR-γ2) and CCAAT/enhancer-binding protein α genes expressed in differentiated adipocytes (C/EBPα) (d), and alkaline phosphatase (ALP) and osteopontin genes expressed in differentiated osteocytes (h). Morphology of differentiated hepatocytes (i). Western blot analyses of albumin (66 kDa) expressions between rat bone marrow-derived cells (rBMC) and rMSC during hepatogenic differentiation (j).

**Characterization of isolated rMSC.** MSC isolated from rat bone marrow were analyzed by FACS and immunocytochemistry for the presence of cell surface markers (Fig. 1). Over 95% of the sorted MSC expressed CD90 (Fig. 1a,e), but not CD34 (Fig. 1b,c). Almost all cells showed adherence dependency, and displayed a spindle-like shape (Fig. 1e,f). Their potential to differentiate into multiple lineages was also verified (Fig. 2). After adipogenic differentiation was induced, intracellular lipid droplets were evident upon Oil Red O staining (Fig. 2a–c). In addition, adipocyte-specific messages, such as transcription factors of peroxisome proliferator-activated receptor-γ2 and CCAAT/enhancer-binding protein-α, were expressed (Fig. 2D). Conversely, during the osteogenic differentiation of isolated rMSC, calcium phosphate depositions were visible following treatment with Tris-maleate solution (Fig. 2e–g), and osteocyte-specific genes of ALP and osteopontin were detected (Fig. 2h).

**Figure 3:**
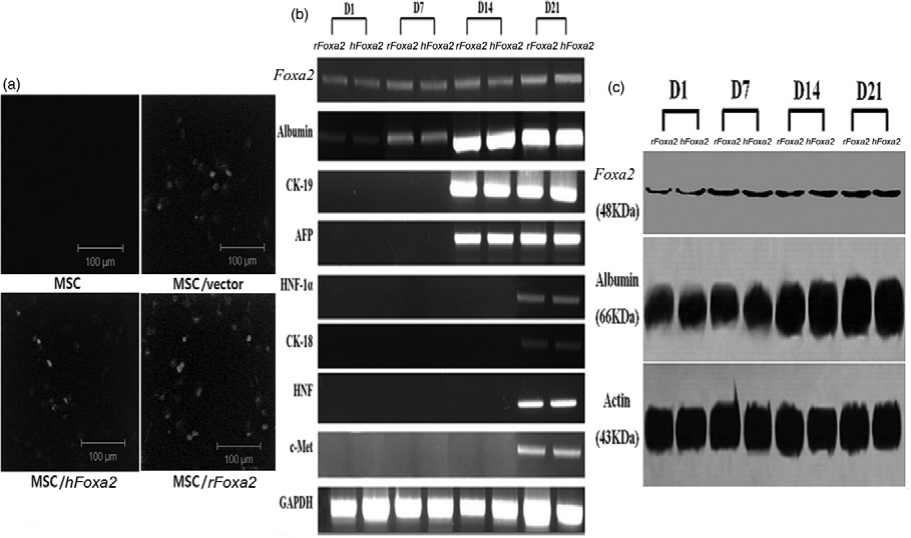
Rat mesenchymal stem cells (rMSC) stably transfected with forkhead box A2 (*Foxa2*) genes and their gene expression profiles following hepatogenic induction. rMSC were transfected with vector (pIRES–enhanced green fluorescent protein [EGFP]) only (upper right), MSC (upper left), or MSC/*Foxa2* (lower left and right) (× 100). (a) Reverse transcription–polymerase chain reaction analyses of liver-specific gene expression in rMSC containing *Foxa2* at days 1, 7, 14, and 21 after the initiation of differentiation. (b) Western blot analysis of the albumin and *Foxa2* in hepatocytes originated from rat *Foxa2* (*rFoxa2*)- and human *Foxa2* (*hFoxa2*)-transfected rMSC (c). AFP, α-fetoprotein; CK, cytokeratin; HNF, hepatocyte nuclear factor.

**Hepatogenic differentiation of rMSC.** rMSC and *Foxa2*-transfected MSC in basal culture medium maintained their spindle-like shape, whereas both cell lines in the hepatogenic differentiation medium gradually changed their morphologies into rounder and smaller compact shapes (Fig. 2i).^22^ The expression of a liver-specific marker, albumin, gradually increased during hepatogenic differentiation (Figs 2j,3).

**Transfection of *hFoxa2* into rMSC.** To use *hFoxa2* in a rat disease model, the expression capability of the *hFoxa2* gene in a pIRES–EGFP expression vector was compared with that of the *rFoxa2* gene in rMSC. After selection with geneticin (G418), respective MSC transfected with the *hFoxa2* gene were green fluorescent protein positive, which was the same as MSC transfected with *rFoxa2* (Fig. 3a). In addition, RT–PCR analyses showed that two gene types induced almost the same degree of expression of hepatoblast markers, including albumin, α-fetoprotein (AFP), cytokeratin (CK)-18, *Foxa2*, HNF, and C-met, during the hepatogenic differentiation process (Fig. 3b). As hepatogenic differentiation progressed, the expression of hepatocyte-specific markers, such as albumin and HNF-3β, increased steadily. Hepatoblast markers, such as CK-19 and AFP, were also expressed, but only during the latter half of the differentiation process. In addition, the expression of liver-specific markers, HNF-1α, CK-18, HNF, and C-met, were detected toward the end of the induction process. Western blot analyses revealed that albumin and *Foxa2* expressions by both gene types (*rFoxa2* and *hFoxa2*) increased steadily upon hepatogenic differentiation induction (Fig. 3C), and their expressions were similar to those of their RNA expressions. Overall, no difference in the hepatogenic differentiation of *rFoxa2* and *hFoxa2* was detected.

**Figure 4:**
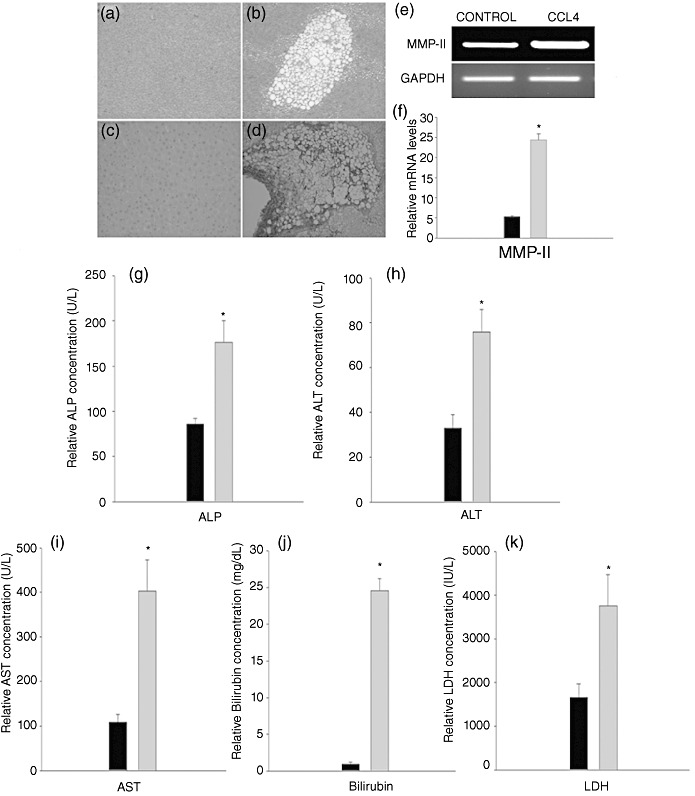
Induction of liver fibrosis in Sprague–Dawley rats by carbon tetrachloride (CCl4). Liver tissues were stained with hematoxylin–eosin and α-smooth muscle actin staining (a,c: control, b,d: CCl4 treated) (×400) and reverse transcription–polymerase chain reaction analyses of matrix metalloproteinase-II (MMP-II) expression in liver tissues in the fibrosis model (e,f). Expressions of several hepatic enzymes in serum were compared (g,k). *Significant difference (*P* < 0.05). (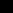) Control, (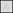) CCl4. ALP, alkaline phosphatase; ALT, alanine aminotransferase; AST, aspartate aminotransferase; LDH, lactate dehydrogenase.

**Induction of the CCl4-injured rat liver fibrosis model.** Chronic liver disease in rats was induced by an intraperitonal injection of CCl4 for 8 weeks, and confirmed by both tissue staining and hepatic enzyme analyses (Fig. 4). The liver tissues of rats treated with CCl4 were morphologically distinguished, and displayed a range of severe injury when compared to that of untreated control rats. As the inflammation increased, fibrosis of the liver developed progressively in certain parts of the liver tissue (Fig. 4a–d). Furthermore, more than twofold higher levels of liver enzymes (AST, ALT, ALP, bilirubin, and LDH) were observed in CCl4-treated rats than in control rats (*P* < 0.05) (Fig. 4g–k).

**Figure 5:**
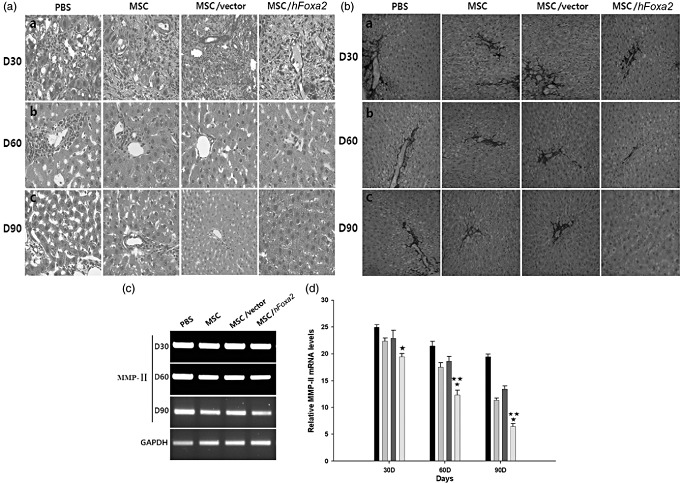
Pathological analyses of the liver tissues. Tissue sections on days 30 (a), 60 (b), and 90 (c) after transplantation were examined by hematoxylin–eosin staining (×400) (a) and by α-smooth muscle actin immunostaining (×100) (b). Matrix metalloproteinase-II (MMP-II) gene expression in reversibility model liver tissues at days 30, 60 and 90 after the transplantation (c,d). *Versus phosphate-buffered saline (PBS); **versus mesenchymal stem cells (MSC) and MSC/vector groups (*P* < 0.05). (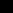) PBS, (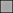) MSC, (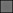) MSC/vector, (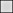) MSC/human forkhead box A2 (*hFoxa2*).

**Transplantation of *hFoxa2*/MSC and histopathology.** To directly verify the efficacy of *hFoxa2* overexpression in treating liver fibrosis, transplantations of different cell types (PBS, MSC, vector only in MSC, and *hFoxa2* gene in MSC) were conducted, and histological analyses were performed. Although all treatment groups exhibited slightly reduced fibrosis areas in the periportal region, there was no difference in the fibrosis area recovered among groups until 30 days after transplantation (Fig. 5a,b). However, on day 60 after post-transplantation, the fibrotic area in the liver decreased significantly in the MSC/*hFoxa2* treatment group (Fig. 5a,b). Furthermore, when compared with either the MSC or MSC/vector group, the fibrotic area was no longer present in the periportal region on day 90 after MSC/*hFoxa2* transplantation (Fig. 5a,b). Immunohistochemical analyses of α-SMA expression were also conducted on sections from all treatment groups after cell transplantation. When compared to other treatment groups, the MSC/*hFoxa*2 treatment group very rarely detected aggregates of cells on day 90 after transplantation, which was similar to the results of the HE staining.

**Figure 6:**
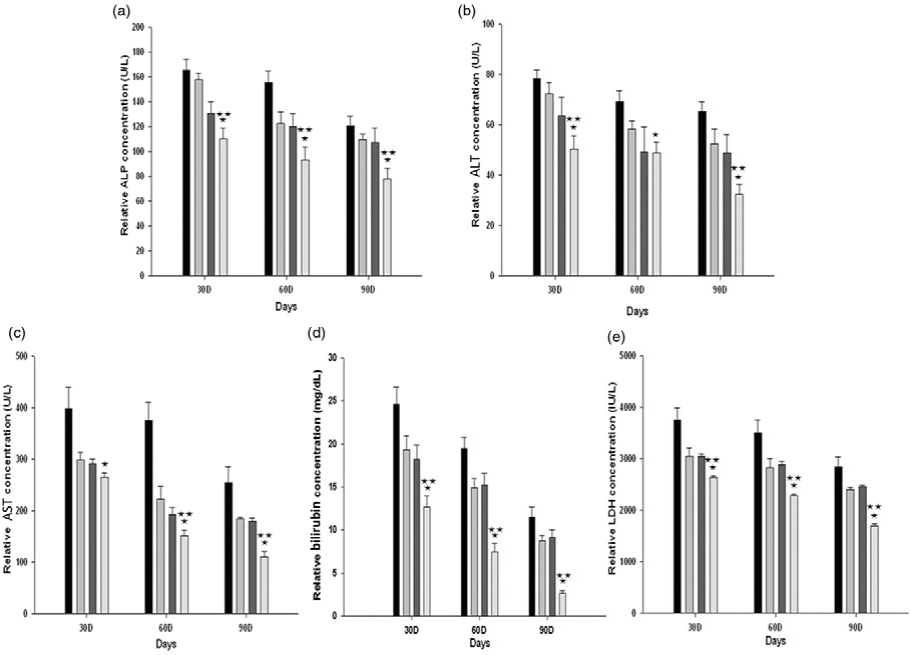
Comparison of serum hepatic enzyme levels after transplantation. Mean values of alkaline phosphatase (ALP) (a), alanine aminotransferase (ALT) (b), aspartate aminotransferase (AST) (c), bilirubin (d), and lactate dehydrogenase (LDH) (e) as a function of post-transplantation on days 30, 60, and 90. *Versus phosphate-buffered saline (PBS); **versus mesenchymal stem cells (MSC) and MSC/vector groups (*P* < 0.05). (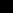) PBS, (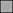) MSC, (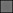)pIRES–enhanced green fluorescent protein/MSC, (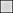) human forkhead box A2 (*hFoxa2*)*/*MSC.

**Expression of liver-specific enzymes after cell transplantation.** As an indirect indicator for the recovery of damaged liver tissues after cell transplantation, serum levels of liver-specific enzymes were measured. All parameters tended to decrease in all treatment groups with time when compared to the PBS control group (Fig. 6). The AST, bilirubin, and LDH levels on all test days were significantly suppressed by cell transplantation (*P* < 0.05), whereas ALT and ALP were not. Among the treatment groups, MSC and vector/MSC showed similar efficacies in the recovery of liver fibrosis. However, the *hFoxa2*/MSC transplantation showed significant effectiveness for liver regeneration, shown as a function of all enzyme levels, as well as a function of days (*P* < 0.05). On day 90 after transplantation, the levels of all enzymes following MSC/*hFoxa2* treatment almost recovered to normal (Fig. 4g–k). In addition, the serum AST levels in the *hFoxa2*/MSC group decreased significantly to approximately half of that in the MSC group (Fig. 6c).

## Discussion

Liver tissue transplantation has commonly been used to treat acute and chronic liver patients,^23^,^24^ but the recent development of various therapeutic modalities enables the potential for use as alternatives to transplantation. Recent rapid developments in stem cell biology have made its use possible in the treatment of several types of diseases.^25^,^26^ In this context, adult stem cells have shown the capacity for regeneration of damaged tissues or organs, including the liver.^27^,^28^ Studies have demonstrated that bone marrow-derived MSC migrate into the damaged liver and contribute to overcoming liver dysfunction.^29^,^30^ However, further investigation is required to increase the efficacy of stem cell-based therapy for the treatment of liver damage. Thus, this study was conducted to examine the regeneration potential of bone marrow-derived MSC modified with the *hFoxa2* gene in the treatment of liver fibrosis induced by CCl4.

As a preliminary study, rat bone marrow-derived cells and MSC were compared to determine whether they differed in their hepatogenic differentiation potentials. After 21 days of hepatogenic induction, it was found that they were not different (Fig. 2). Thus, MSC were used for further studies due to the homogenous population (Fig. 1).

The potential of bone marrow-derived MSC to differentiate into other lineages was morphologically and biochemically verified (Fig. 2), which is consistent with the results of other reports.^31^,^32^ Under hepatogenic induction conditions, MSC gradually exhibited morphological changes from a spindle shape into a characteristic compact, polygonal shape, and then began to clump (Figs 2,3), in accordance with previous findings.^33^^–^^35^

Although MSC represent a promising source of autologous cells for stem cell therapies,^36^,^37^ the success of MSC engraftment has been extremely low. The degree of therapeutic effects might be affected by various factors, such as cell type, transplant method, disease model, and treatment condition.^38^,^39^ Recent studies have demonstrated that the application of transcription factors and growth factors, such as HNF-3β, insulin-like growth factor-1, and HGF, contributed to the recovery of damaged liver tissues, owing to their capabilities to induce hepatoprotective factors.^40^,^41^ In particular, *Foxa2* (HNF-3β) is a transcriptional activator for liver-specific genes, which is involved primarily in hepatic metabolism, as well as early developmental events.^42^ Therefore, we applied the *Foxa2* gene to improve the MSC strategy for hepatogenesis.

It has been reported that *rFoxa2* and *hFoxa2* share more than 96.4% homology in amino acid sequences, and the winged-helix DNA-binding domains of the two genes are perfectly conserved.^43^ As expected, the genes were not different with respect to the expression of liver-specific markers, including albumin, AFP, and CK-18 (Fig. 3). Therefore, *hFoxa2* was cloned into the pIRES–EGFP expression vector, which will be an expression system for preclinical studies in the future. MSC transfected with *hFoxa2* were also confirmed for their differentiation potential to hepatocytes.

The therapeutic capacities of undifferentiated MSC with or without gene modification were tested (Figs 5,6). Histopathological analyses revealed that transplanted rMSC overexpressing *hFoxa2* did not show any acute recovery of damaged liver tissue at the beginning of post-transplantation. However, rMSC with or without *hFoxa2* began to affect damaged tissue after approximately 30 days, which differed from the PBS control group, indicating that cells were engrafted and differentiated. These findings are consistent with those of a previous study, in which cells were observed in the sinusoid for 1 week after transplantation, migrated into liver tissue 1 week after engraftment, and then took 2 weeks to differentiate into hepatocyte-like cells.^19^,^22^ Importantly, when compared to the fibrotic areas of the samples treated with MSC alone or MSC/vector, the fibrotic area of the samples treated with rMSC/*hFoxa2* for 90 days significantly decreased, until they were completely gone (Fig. 5a). These results support previous findings that *Foxa2* interacts with the enhancer region of the albumin gene and accelerates hepatogenic differentiation of MSC in damaged liver tissue.^44^

When those specimens were immunostained with the α-SMA antibody, the same results were obtained (Fig. 5b). As smooth muscle cells interact with extracellular matrix components, notably collagen, this indirect observation implies important roles of *hFoxa2* in the recovery of damaged liver tissue. Previous reports have suggested that liver fibrosis might be largely relevant to the deposition of collagen. Therefore, the matrix metalloproteinase (MMP) family, such as MMP-II, should influence the degradation of collagen.^45^,^46^ Our results are consistent with those of previous studies, showing that MMP-II decreased gradually during tissue regeneration (Figs 4,5). In addition, biochemical analyses of liver-specific enzymes decreased at 60 days' post-transplantation in all treatment groups, but a group of animals treated with MSC/*hFoxa2* showed significant recovery of those enzyme expressions to normal levels at the end of the study (90 days). Furthermore, they did not show any signs of tumor development or of other side-effects, even a couple of months after completion of this experiment (data not shown).

Overall, the distinct difference in the recovery of damaged tissue obtained from this study indicates that *hFoxa2* efficiently promoted the incorporation of MSC into liver grafts, and suggests that the effects of *hFoxa2* might be attributed to the stimulation of the expression of antifibrotic factors.

Several approaches using various types of stem cells to treat liver disease are currently being investigated, including the use of embryonic stem cells and induced pluripotent stem cells.^47^,^48^ However, it is generally accepted that those need to overcome many technical difficulties of low efficiency and to clarify long-term effects. In that context, a combination of gene therapy and cell therapy might be considered an effective therapeutic approach at the present time. Thus, the present study of gene-modified therapy using the *hFoxa2* gene in MSC is suggested to be an effective approach.
